# The Road so Far in Colorectal Cancer Pharmacogenomics: Are We Closer to Individualised Treatment?

**DOI:** 10.3390/jpm10040237

**Published:** 2020-11-19

**Authors:** Ana Rita Simões, Ceres Fernández-Rozadilla, Olalla Maroñas, Ángel Carracedo

**Affiliations:** 1 Grupo de Medicina Xenómica, Universidade de Santiago de Compostela (USC), 15706 Santiago de Compostela, Spain; anarita.santos.simoes@rai.usc.es (A.R.S.); olalla.maronas@usc.es (O.M.); angel.carracedo@usc.es (Á.C.); 2 Instituto de Investigación Sanitaria de Santiago (IDIS), 15706 Santiago de Compostela, Spain; 3 Fundación Pública Galega de Medicina Xenómica; SERGAS, 15706 Santiago de Compostela, Spain; 4 Consorcio Centro de Investigación Biomédica en Red de Enfermedades Raras—CIBERER, 28029 Madrid, Spain

**Keywords:** colorectal cancer, adverse drug reactions, pharmacogenomics, personalised medicine, toxicity

## Abstract

In recent decades, survival rates in colorectal cancer have improved greatly due to pharmacological treatment. However, many patients end up developing adverse drug reactions that can be severe or even life threatening, and that affect their quality of life. These remain a limitation, as they may force dose reduction or treatment discontinuation, diminishing treatment efficacy. From candidate gene approaches to genome-wide analysis, pharmacogenomic knowledge has advanced greatly, yet there is still huge and unexploited potential in the use of novel technologies such as next-generation sequencing strategies. This review summarises the road of colorectal cancer pharmacogenomics so far, presents considerations and directions to be taken for further works and discusses the path towards implementation into clinical practice.

## 1. Introduction

Colorectal cancer (CRC) is the second leading cause of cancer-related death and the third most commonly diagnosed cancer [1]. Surgical resection is the preferable treatment independently of stage, but chemotherapy is widely used too across stages. There are different chemotherapeutic schemes for CRC treatment (Table 1).

Usually, the first line of treatment is based on fluoropyrimidines: 5-fluorouracil (5-FU) or its oral prodrug capecitabine, either alone or in different combinations with other agents, the most common being leucovorin, oxaliplatin (named FOLFOX or XELOX -if capecitabine is used instead of 5FU) or irinotecan (FOLFIRI) [2,3,4,5]. Besides these cytotoxic agents, metastatic CRC (mCRC) treatment may in addition include biological targeted agents to improve patient outcome, such as monoclonal antibodies against vascular endothelial growth factor (VEGF) (bevacizumab), or against epidermal growth factor receptor (EGFR) (cetuximab and panitumumab) (Table 1) [4].

There are two essential factors to be taken into account when considering efficacy and appropriateness of a treatment: response and toxicity. Response is often evaluated based on overall survival, progression-free survival or response evaluation criteria in solid tumors (RECIST), in the case of unresectable CRC [6]. On the other hand, patients subject to chemotherapy are prone to develop adverse drug reactions (ADRs) that might be severe or even fatal, and have a considerable impact on healthcare and burden. These ADRs can affect the patients’ quality of life (even in the long term) and may hinder treatment, due to necessary delays or dose reduction. A study with more than four thousand mCRC patients receiving FOLFOX, FOLFIRI or XELOX saw that 90% of patients had one ADR, and 66% of patients had >1 ADR during the first line of treatment [7]. These toxic events also come with an increased economic burden to resolve them, with haematological toxicities being the most costly to resolve, followed by respiratory, endocrine/metabolic, central nervous system and cardiovascular ones.

Since both response and toxicity events have heterogeneous distributions amongst patients, it has been hypothesised that these ADRs may be caused by underlying genetic variants. Moreover, because chemotherapy agents have only been used since the 1950s, any genomic variants having large effects on toxicity responses have not had time to be washed away by negative selection [8,9]. Moreover, because cancer is usually related to later stages of life and does not affect fitness, purifying selection against these variants is not in place. Therefore, it is feasible that genetic variants having moderate-to-large effects (detectable by classical association studies) could be responsible for the observed variability.

Pharmacogenetics is a science that aims to learn about the inherited inter-variability in response and ADRs after drug exposure. First-generation studies were focused on the analysis of genes with an a priori relationship to drug effect, i.e., those involved mainly in the adsorption, distribution, metabolism and excretion (ADME) of chemotherapeutic agents. Later, these studies started to apply global approaches without a previous functional hypothesis, like genome-wide association studies (GWAS). The Pharmacogenomics Knowledgebase (PharmGKB [10]) is a free database that aggregates, curates, integrates and disseminates the knowledge obtained from these studies regarding the impact of human genetic variation on drug response and toxicity. Other important sources of pharmacogenomic information have also launched from the efforts of The Clinical Pharmacogenetics Implementation Consortium (CPIC), which aims to create, curate and post free detailed gene/drug clinical practice guidelines (https://cpicpgx.org/ (accessed on 29 October 2020)).

In this review, we summarise the available data on CRC pharmacogenomics to date and go beyond the typically discussed candidate gene approaches, to cover genome-wide studies and next-generation sequencing. We also reflect on the necessity of comprehensive works including molecular studies to assess variant functionality, and discuss the limitations towards clinical implementation in the light of cost-effectiveness to health systems. Last but not least, we discuss considerations for further studies towards a routine implementation of personalised medicine strategies in clinical practice.

## 2. Chemotherapeutic Agents in CRC Treatment

**Figure 1:**
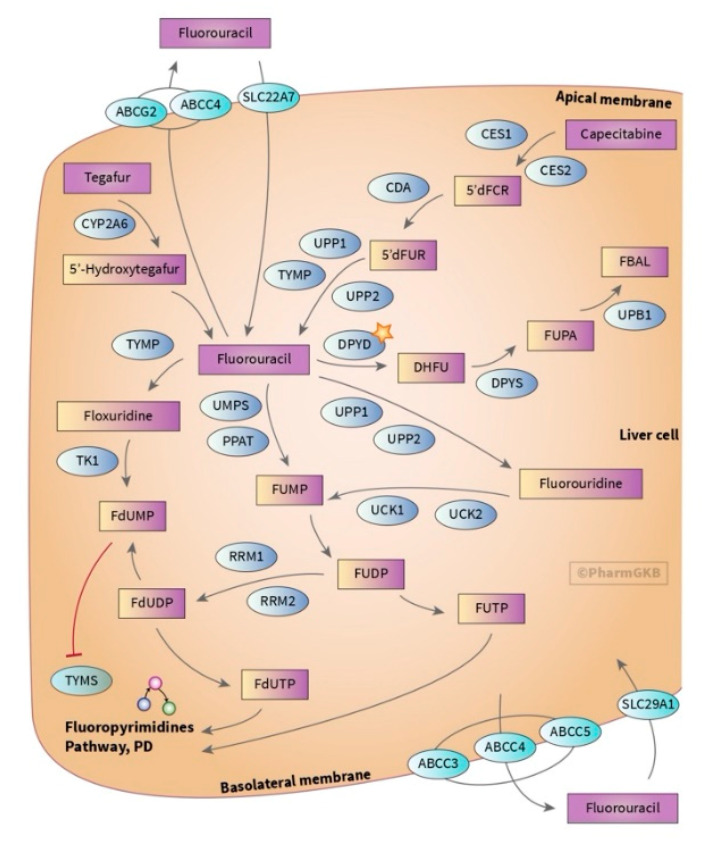
Graphic scheme of the genes involved in the adsorption, distribution, metabolism and excretion (ADME) of fluoropyrimidines [20]. Capecitabine passes through the gut wall and is metabolised into 5-deoxyfluorocytidine (5′dFCR) and 5’-deoxy-5-fluorouridine (5′dFUR) by carboxyl esterases (CES) and cytidine deaminase (CDA), respectively, and activated into 5-FU by thymidine phosphorylase (TP). - 5-FU is metabolised mostly in the liver by dihydropyrimidine dehydrogenase (DPD) (<80%) into dihydrofluorouracil (DHFU). The secondary elimination pathway is through urinary excretion or catabolism in extrahepatic tissues [21]. Its mechanism of action involves the methylenetetrahydrofolate reductase (MTHFR)—converting 5,10-methylentetrahydrofolate (5,10-MTHF) into 5-MTHF, which is required for purine and thymidine synthesis, and thymidylate synthase (TS) enzymes—forming a complex with 5,10-MTHF and deoxyuridine monophosphate (dUMP), which in the end disrupts DNA replication and repair. Used with PharmGKB and Stanford University permission (available at https://www.pharmgkb.org/pathway/PA150653776 (accessed on 24 September 2020)).

Chemotherapy based on fluoropyrimidines, specifically 5-FU, has been used for over thirty years now, and is still the backbone of CRC treatment (Figure 1) [11]. However, there have been reports that show that up to 94% of patients treated with this drug end up developing ADRs, some of which may be severe or life threatening (Table 2) [12]. For instance, some studies have shown that around 40–56% of patients treated with 5-FU develop severe neutropenia, and 10–15% present grade 3–4 diarrhoea [13]. Patients receiving capecitabine have a similar incidence of ADRs, although with less severe neutropenia, but present hand–foot syndrome (HFS) at a high incidence (54%) instead (Table 2) [14].

**Figure 2:**
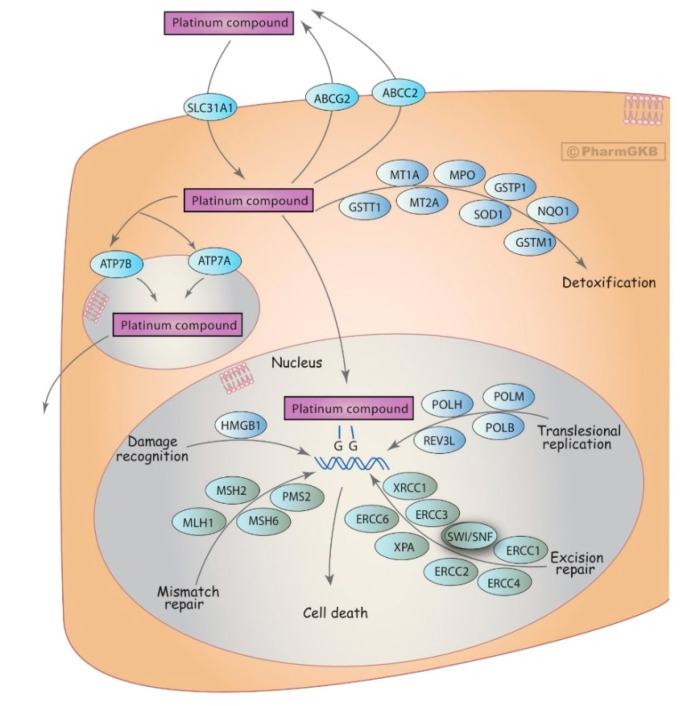
Graphic scheme of the genes involved in the ADME of platinum compounds, including oxaliplatin [23]. The glutathione S-transferases (GSTs), a multigene family of enzymes, undertake oxaliplatin detoxification. The solute carriers (SLCs) and adenosine-triphosphate binding cassette (ABC) transporters are responsible for oxaliplatin uptake and efflux in the liver, respectively, and so impact on drug bioavailability and toxicity profile. Further, the nucleotide excision repair (NER) and base excision repair (BER) pathways, which include the ERCC1 and ERCC2, and XRCC1 proteins, respectively, repair the damages cause by this drug. Used with PharmGKB and Stanford University permission (available at https://www.pharmgkb.org/pathway/PA150642262 (accessed on 24 September 2020)).

Platinum-based drugs, mainly oxaliplatin, are cytotoxic agents that prevent neoplastic proliferation, by forming DNA–platinum adducts, which block replication and transcription and induce apoptosis (Figure 2) (Table 1) [22]. The main oxaliplatin dose-limiting toxicity is neuropathy, occurring in about >90% of treated patients (Table 2) [15].

**Figure 3:**
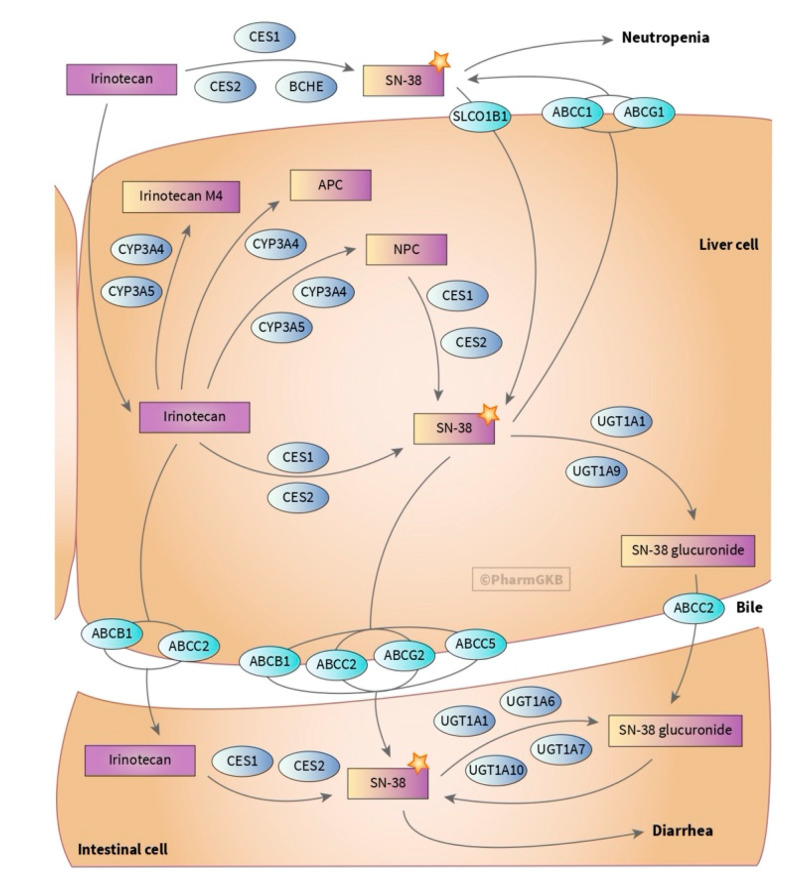
Graphic scheme of the genes involved in the ADME of irinotecan [10]. Irinotecan is converted into SN-38 by CES, which inhibits topoisomerase I, an enzyme essential for DNA replication and then into inactive SN-38G by UGTs. Further, it can suffer oxidation into 7-ethyl-10-[4-N-(5-aminopentanoic acid)-1-piperidino] carbonyloxycamptothecin (APC), M4 and 7-ethyl-10-[4-(1-piperidino)-1-amino] carbonyloxycamptothecin (NPC) by CYP3A4 and CYP3A5. NPC can be reactivated by CES into SN-38. Irinotecan and its metabolites’ uptake and efflux are conducted by SLCs and ABC transporters, respectively. Used with PharmGKB and Stanford University permission (available at https://www.pharmgkb.org/pathway/PA2001 (accessed on 24 September 2020)).

Irinotecan (CPT-11) is another cytotoxic agent used in the treatment of CRC in combination with 5-FU (FOLFIRI) (Table 1). FOLFIRI treatments result in better response rates and longer progression-free survival and overall survival of mCRC patients (Figure 3) [2,24]. CPT-11 is a semi-synthetic soluble analogue of the natural alkaloid camptothecin [25,26]. Some clinical trials report an ADR incidence for this drug of up to 100% of patients, where common ADRs include diarrhoea, neutropenia and alopecia (Table 2) [16].

In case of unresectable CRC, patients may also be given biological targeted agents. Cetuximab and panitumumab bind specifically to the human EGFR protein, which is constitutively expressed in normal epithelial tissues and overexpressed in some cancers like CRC. Some of the pioneer pharmacogenetics studies on treatment efficacy found, however, that because *RAS* mutations can constitutively activate the response pathway downstream from EGFR, anti-EGFR therapy efficacy is limited to patients’ wild type for *KRAS* and *NRAS* [4]. These belong to signalling pathways downstream of EGFR, and mutations in these genes may cause EGFR-independent pathway activation, leading to resistance to anti-EGFR treatments [27]. More than 87% of patients receiving cetuximab develop an ADR and are commonly (>25%) prone to develop cutaneous reactions, headache, diarrhoea and infection, whereas patients receiving panitumumab (>20%) will probably have cutaneous reactions, fatigue, nausea and diarrhoea [17,18,25,26]. On the other hand, bevacizumab binding to VEGF blocks the interactions with its receptors on the endothelial cell surface. This interaction allows cell proliferation and angiogenesis, and thus bevacizumab reduces microvascular growth and inhibits metastatic progression. Over 60% of patients receiving bevacizumab develop ADRs, where the most common are hypertension, proteinuria, mucosal bleeding and wound healing problems [4,19].

## 3. Pharmacogenetics: Candidate Gene Studies

As we mentioned before, pharmacogenetic studies arose in the context of studying the genetic factors that contribute to ADRs. Initial efforts utilised candidate gene approaches to inspect mainly genetic variation in genes that might have a great influence on the drug pharmacokinetics and pharmacodynamics, and that can alter drug concentration levels, leading to toxicity.

### 3.1. Dihydropyrimidine Dehydrogenase (DPYD)

DPD, encoded by the *DPYD* gene, is responsible for the vast majority of 5-FU hepatic metabolism and is responsible for the first step and rate-limiting factor in the 5-FU catabolic pathway (Figure 1). Several single nucleotide polymorphisms (SNPs) have so far been identified in this gene in association with different toxicities [28]. The most studied *DPYD* variant is rs3918290 (DPYD*2A, IVS14+1G>A), which causes exon 14 skipping and results in a truncated and catalytically inactive protein [29,30]. A study by Toffoli et al. on 603 patients treated with 5-FU-based chemotherapy reported the association of rs3918290 (OR = 8.5, *p* = 0.008), rs67376798 (OR = 7.8, *p* = 0.012) and rs55886062 (OR = 6.0, *p* = 0.131) with general toxicity (Table 3) [28].

A further meta-analysis including 7365 patients from eight different studies confirmed the association between *DPYD* rs55886062 (DDYD*13) and *DPYD* rs56038477 with gastrointestinal (OR = 5.72, *p* = 0.015; 2.04, *p* < 0.0001, respectively) and haematological toxicities (OR = 9.76, *p* = 0.00014; and 2.07, *p* = 0.013, respectively), and also between *DPYD* rs3918290 and rs67376798 with overall toxicity (OR = 20.5, *p* < 0.0001; and 3.02, *p* < 0.0001, respectively) [34].

### 3.2. Thymidylate Synthetase (TYMS)

TS, encoded by the *TYMS* gene, is the main target of fluoropyrimidines and low levels of expression may influence toxicity [86,87]. The two most studied SNPs in *TYMS* are rs2853542 (5′VNTR 2R/3R) and rs11280056 (3′UTR 6bp ins-del). This gene has been widely studied, but with no conclusive results so far. Some studies have reported a correlation between rs2853542 and 5-FU/capecitabine toxicity, where the haplotype 2R/ins 6-bp was found to be significantly associated with severe toxicity [45,87], but other works could not replicate this association [61]. This might be explained by a work of Rosmarin et al. in 2015, which reported an association of an intronic variant located in the overlapping *ENOSF1* gene capable of explaining the toxicity attributed to the two previous *TYMS* polymorphisms. They discovered that SNP rs2612091 and *TYMS* 5′VNTR and 3′UTR are in moderate linkage disequilibrium (LD) (r^2^ = 0.40 and 0.32, respectively), but after testing for dependency, they concluded that it was the rs2612091 G allele alone that increased the risk of toxicity (*p* = 0.0021). Although it has been proposed that the ENOSF1 protein could influence TYMS activity, the interaction between these two genes is not yet well understood [41]. Interestingly, genetic variation in *TYMS* has also been related to response to pyrimidine treatments, with higher levels of TS implicating worse response and poorer overall survival [88,89].

### 3.3. Methylenetetrahydrofolate Reductase (MTHFR)

MTHFR is the other major enzyme involved in 5-FU metabolism. Polymorphisms in this gene (namely rs1801133 and rs1801131) might impact enzyme activity, causing an accumulation of 5,10-MTHF, which increases toxicity [90]. Indeed, a study involving 292 stage II/III colon cancer patients found that the rs1801133 TT genotype was associated with neutropenia (OR = 2.32, *p* = 0.014) [48]. Another study involving 118 mCRC patients found that the same genotype was associated with diarrhoea (*p* = 0.02) [91]. However, other studies have not been able to find any association between polymorphisms in this gene and toxicity events [61,62,92,93].

### 3.4. Carboxyl Esterases (CES) and Cytidine Deaminase (CDA)

CES2 is the first enzyme in the conversion of capecitabine to 5-FU, followed by a second step catalysed by CDA (Figure 1). There have been some attempts to prove the association of polymorphisms on these two genes with ADRs, but there are still no concrete positive results. Ribelles et al. studied 136 patients and showed a trend (*p* = 0.07) between HFS and *CDA* SNP rs3215400 [54]. A study including 239 patients found an association of *CDA* rs2072671 with a high risk of overall toxicity (OR= 1.84, *p* = 0.029) [53]. Another work including 430 patients linked the *CDA* rs602950 and *CDA* rs532545 variants with diarrhoea (OR = 2.3, *p* = 0.0055, and 2.3, *p* = 0.0082, respectively) [47]. There have also been some smaller studies on *CES* polymorphisms and their association with capecitabine toxicity [45,54,94]. CES proteins are also important in the catabolic pathway of irinotecan (Figure 3) [95]. *CES1* rs2244613 was found to be associated with diarrhoea and patients with low *CES2* expression are more prone to develop neutropenia or diarrhoea [95,96,97,98].

### 3.5. DNA Repair Genes

DNA repair pathways have been extensively studied in pharmacogenomic studies [99]. A meta-analysis of more than 1000 CRC patients receiving oxaliplatin found a single significant association of the *ERCC1* rs11615 C allele with a higher risk of having haematological toxicity in Asian populations (HR = 1.97, *p* < 0.05) [100]. Boige et al. could not, however, replicate this association, perhaps due to population differences, but did associate the *ERCC2* rs13181 C allele with a higher risk of severe haematological toxicity caused by FOLFOX (OR = 2.16, *p* = 0.01) [62]. A recent study on 596 CRC patients found that *ERCC1* rs11615 was significantly associated with stomatitis (*p* = 0.03) and nausea (*p* = 0.04), and that *ERCC2* rs13181 and rs238406 were associated with thrombocytopenia (*p* = 0.004 and *p* = 0.03, respectively) [63]. On the other hand, a study of 517 patients with stage II/III colon cancer concluded that polymorphisms in *ERCC1* and *XRCC1* did not have a clinically significant association with adverse effects [61]. Further smaller studies could neither confirm the relationship between these variants and toxicity [91,101].

### 3.6. Glutathione S-Transferases (GSTs)

GST enzymes are proteins from a multigene family, and specifically, GSTP1, GSTM1 and GSTT1 are involved in oxaliplatin detoxification (Figure 2). The most studied variations are *GSTP1* rs1695 and the complete deletion of the *GSTT1* and *GSTM1* genes. McLeod et al. tested these on 300 patients receiving FOLFOX in an advanced CRC setting. Patients bearing the *GSTM1* null genotype had a 1.7-fold increased risk of having severe neutropenia (*p* = 0.016), whereas homozygous patients for the rs1695 T allele had higher probability of discontinuing FOLFOX treatment due to neurotoxicity (*p* = 0.01) [102]. In contrast to these findings, Boige et al. did not find any significant association between these same SNPs and severe neurotoxicity on a study enrolling 349 patients [62]. Ruzzo et al. studied 517 patients and suggested a weak association between the *GST-T1/M1* null/null genotype and severe neutropenia (OR = 1.99, *p* = 0.032) [61], whereas Cecchin et al. analysed 154 patients receiving FOLFOX but could not replicate any markers of neurotoxicity. Interestingly, they suggested that variants other than genetics, such as the biological state of patients or disease stage, may also influence the detoxification pathway, and could therefore be responsible for the FOLFOX-related neurotoxicity [58].

### 3.7. Adenosine-Triphosphate Binding Cassette (ABC) Transporters

Genes within the ABC transporter family are responsible for the efflux of a variety of drugs and their metabolites, including oxaliplatin and irinotecan. However, there is a lot of controversy on the relationship of polymorphisms on *ABC* genes and chemotherapy-related toxicity. For 206 patients receiving FOLFOX, Custodio et al. reported that the *ABCG2* rs3114018 AA genotype had a significantly higher risk of neuropathy (OR = 2.67, *p* = 0.059) [59]. In a study including 144 patients, Cecchin et al. reported positive associations with neurotoxicity for SNPs in *ABCC2*: rs3740066 (OR = 2.99, *p* = 0.0231), rs1885301 (OR = 3.06, *p* = 0.0072), rs4148396 (OR = 4.69, *p* = 0.0048) and rs717620 (OR = 14.39, *p* = 0.0164), which are in high LD with one another. Others studies have been less successful in linking genetic variants in this gene with neurotoxicity or other toxicities [58,61,103].

In relation to irinotecan-based regimens, Salvador-Martín et al. showed that SNPs rs1128503, rs2032582 and rs1045642 in *ABCB1*, which are in LD, were associated with haematological and overall toxicity [92]. Others proposed the association of solely *ABCB1* rs1128503 (OR = 2.02, *p* = 0.401) with global toxicity, or of *ABCB1* rs1045642 with early toxicity (OR = 3.79, *p* = 0.098) (not strictly significant), while others did not find any association at all [74,93,95,96]. There have also been some reports on other *ABC transporter* genes, with conflicting results. For instance, a study on 26 mCRC patients showed that patients with the CC genotype in *ABCC5* rs562 or the GG genotype in *ABCG1* rs425215 presented higher gastrointestinal toxicity (*p* < 0.02) [72]. A study including 250 patients with mCRC linked the *ABCG2* rs7699188 variant with severe global toxicity (OR = 7.26, *p* = 0.013) [74].

### 3.8. Uridine Disphosphate Glucuronosyltransferases (UGTs)

UGT1A1 is the main enzyme responsible for SN-38 inactivation, followed by UGT1A7 and UGT1A9. Several groups have studied the influence of *UGT* polymorphisms on toxicity development. One of the most studied polymorphisms in *UGT1A1* is a change in the number of TA repeats (TA)_n_TAA in the promoter region. The wild-type allele for this polymorphism is (TA)_6_TAA, with (TA)_7_TAA (rs3064744, UGT1A1*28) being frequent in Caucasians, but not in Asian populations (≈30% and ≈10%, respectively). However, rs4148323 (UGT1A1*6) is more frequent in Asian populations comparing with Caucasians (≈14% and ≈1%, respectively). Ando and colleagues reported that patients carrying the UGT1A1*28 genotype were at significantly higher risk of having irinotecan-related severe toxicity (OR = 7.23, *p* < 0.001) [64]. Innocenti et al. also stated that patients with UGT1A1*28 had more events of severe neutropenia (OR = 9.3, *p* = 0.001) [67]. Others have also showed a correlation between UGT1A1*28 and neutropenia, diarrhoea and vomiting (*p* < 0.01) [104,105,106,107]. Additionally, as for *TYMS*, it has been proven that the *UGT1A1* genotype also affects maximum tolerated dose and therefore response [108,109].

### 3.9. Solute Carriers (SLCs)

Reduction or elimination of the function of SLC genes due to genetic variation can lead to a decrease in SN-38 uptake, with further accumulation in plasma, ultimately leading to toxicity [97]. rs2306283 (SLCO1B1*1b) has been shown to cause severe gastrointestinal toxicity, particularly diarrhoea and neutropenia [72,110,111]. A discovery study on 167 mCRC patients receiving irinotecan also revealed a protective effect of the *SLCO1B1* rs2291076 T allele against neutropenia but associated the rs2306283 GG genotype with significantly higher neutropenia events. These results were, however, not replicated in a posterior study of 250 mCRC patients [71].

### 3.10. Cytochrome p Gene Family (CYP)

CYP3A4 and CYP3A5 are responsible for the oxidation of irinotecan into the inactive metabolites APC, M4 and NPC. Some researchers have studied the possible association of polymorphisms on these genes and chemo-related toxicity but have not found any positive correlation [68,96,112], probably because over 80% of variants in *CYP* genes coding regions are very rare and the sample sizes of these studies were not large enough [113]. It has also been suggested that their enzymatic function might be altered by non-genetic factors such as diet, concomitant medications, altered liver function or patient’s performance status [114].

### 3.11. Epidermal Growth Factor Receptor (EGFR)

Skin toxicity is the major ADR related to anti-EGFR agents. Parmar et al. studied 109 cancer patients and concluded that skin toxicity was linked to the *EGFR* rs2227983 GG genotype (OR = 3.24, *p* = 0.014) [80]. Dahan et al. studied 58 patients treated with third-line cetuximab and irinotecan, and reported a trend between the presence of rs11568315 (CA repeats ≤ 35) and skin toxicity (OR = 2.91, *p* = 0.058) [81]. Sunakawa et al. studied 77 patients treated with cetuximab in combination with oxaliplatin and also correlated rs11568315 (CA repeats ≤ 19) with skin toxicity [115]. A study on 52 patients treated with cetuximab and FOLFIRI found that *EGFR* rs712830 was significantly associated with severe global toxicity (OR = 6.13, *p* = 0.010), but not specifically with skin toxicity. rs712829, rs11568315 (CA repeats cut-off = 17) and rs4444903 were, however, not associated with any toxicity [79]. Another study on 46 mCRC patients receiving XELOX-bevacizumab with or without cetuximab also found no evidence for the association of either rs4444903 or rs11568315 (CA repeats cut-off = 20) with skin toxicity [116].

### 3.12. Vascular Endothelial Growth Factor (VEGF)

Hypertension is the major toxicity derived from anti-VEGF agent treatment. Studies on the relationship of *VEGF* polymorphisms and bevacizumab-related toxicity have also been controversial. For instance, a study on 89 patients reported a positive link between rs3025039 and hypertension (OR = 0.15, *p* = 0.022), but a meta-analysis of over 1000 cancer patients did not validate this finding [83,117]. Moreover, some researchers have reported that patients with the rs833061 TT, rs2010963 CC or rs699947 CC genotypes were less prone to hypertension caused by bevacizumab (*p* < 0.03) [84,85], but Etienne-Grimaldi et al. saw that patients harbouring the rs2010963 CC genotype alone had more toxicity than patients with other genotypes (*p* = 0.01) [118].

### 3.13. Immunotherapy and Toxicity

Immunotherapy has arisen in the past few years as a promising therapeutic option in many cancers, and has particular relevance in the case of tumours with microsatellite instability (MSI) [119]. Hence, the FDA approved, in 2018, the use of ipilimumab and nivolumab (anti-CTLA-4 and anti-PD1 monoclonal antibodies, respectively) for the treatment of metastatic CRC patients previously treated with standard chemotherapy [120]. In 2020, pembrolizumab (anti–PD-1) was also approved as a first-line treatment of patients with unresectable, MSI-high or mismatch repair-deficient metastatic CRC [121]. Although there have been some studies suggesting the influence of genetic variants on the development of toxicity due to these treatments in other cancer types, to date there is no sufficient data on CRC [122,123,124]. Surely novel data on this will shortly become available for pharmacogenomic studies as more patients undergo immunotherapy treatment.

## 4. Pharmacogenomic Approaches

### 4.1. Genome-Wide Association Studies (GWAS)

Despite the large effect sizes for toxicity variants discovered by candidate gene approaches, chemotherapy-related toxicity is likely complex and multigenic. Therefore, other discovery strategies may be more suitable to inspect genomic variation in a more comprehensive manner. This has been made possible by the increasing availability of higher-throughput technologies at increasingly affordable prices, which has allowed pharmacogenetics to go genomic. In these upcoming sections, we will describe the more recent approaches that have further expanded the knowledge on pharmacogenomics in recent years (Table 4).

GWAS make use of LD inheritance patterns to inspect common genetic variation across the entire genome. The main two advantages of GWAS over candidate gene studies are that they are unbiased by a priori functional knowledge on the variants (which may help in the discovery of other toxicity relevant pathways) and also have the potential to identify variation in regulatory regions such as promoters or enhancers, which have been largely unexplored by candidate gene approaches.

Several GWAS have been performed to inspect chemotherapy-related toxicity in CRC. In the QUASAR2 trial, Rosmarin et al. analysed over 1000 stage II/III CRC patients receiving capecitabine with or without bevacizumab to identify 1456 variants on 25 candidate genes (Table 3) [41]. Fernandez-Rozadilla et al. used 1012 patients in a two-stage study in patients treated with 5-FU and FOLFOX [57] to find a moderate association for the rs10876844 variant and diarrhoea in patients treated with 5-FU. Won et al. also completed a GWAS on 343 Korean patients receiving oxaliplatin-based regimens to identify possible genetic markers associated with chronic oxaliplatin-induced peripheral neurotoxicity (OXCPN) [78]. They found some evidence for an association that was intronic or within 100 Kb of genes related to various neuronal activities. Two subsequent and independent studies by Oguri et al. and Terrazzino et al. tried to validate these findings, but a single association between the *FARS2* rs17140129 G allele and OXCPN (OR = 6.5, *p* = 0.034) was found [125,126]. Lastly, the CAIRO2 trial included 282 advanced or metastatic CRC individuals treated with XELOX plus bevacizumab and cetuximab. They found some novel SNPs to be moderately associated with toxicity (Table 3) [82].

In general, although GWAS present several advantages over candidate gene strategies, there are also some important limitations, some of which could be overcome post hoc. Firstly, there is a lack of replication due to discrepancies in variant frequencies amongst the different populations used between studies, as seen when comparing the works from Won et al. and Terrazzino et al. mentioned above (Asian vs. Caucasian populations, respectively). Further, most of the associated variants are intergenic, which makes it harder to interpret the results directly and design appropriate validatory functional assays. Moreover, because we are evaluating thousands to millions of variants at a time, statistical power is a concern, and adequate study sample sizes are needed [127]. As an illustration, for a GWAS with a sample size of 200 patients, assessing variants with minor allele frequency (MAF) ≥ 5%, and a statistical threshold of 80% power, the OR that we would be able to discover is OR ≈ 2, which reflects a moderate effect.

GWAS are limited to inspecting common variation (i.e., generally over 5% MAF), but it is likely that toxicity variants may be of rarer prevalence [128,129]. Some approaches have been developed to overcome this limitation. For instance, targeted SNP panels can be designed to fine-map regions of interest spanning a large section of the gene or specific to a desired population. As an example, a commercially available array has been designed to include both common and low-frequency variation as well as Mendelian and functional alleles specific to Spanish genomes, which allows for better genotyping of the Spanish population when comparing with the generic global arrays [130]. Moreover, albeit possible, GWAS strategies are not usually suitable for CNV studies, because they demand that the CNV be in high LD with a genotyped SNP [57].

Despite these limitations, GWAS still hold great potential for discovery, given appropriate study conditions. Surely, there are still pathways contributing to toxicity development to be discovered, as proven by the contribution of *RPS7* to cetuximab-related toxicity. This gene is normally overexpressed in dermal papilla cells, which makes it reasonable that genetic variants could be associated with skin toxicity [82].

### 4.2. Next-Generation Sequencing (NGS)

NGS, either whole-exome (WES) or whole-genome sequencing (WGS), allows for a more comprehensive identification of novel genetic biomarkers in this regard, and several studies have reported the added value of NGS to identify relevant rare pharmacogenetic variants that would not be detected by other conventional methods (Table 4) [131,132,133,134,135,136].

In 2014, Mizzi et al. compared the data from 482 healthy individuals (data from Genomes Data and the Wellderly Study) obtained either with WGS or SNP array genotyping that included 1936 known pharmacogenomic variants within 231 ADMET genes (Table 5) [131]. Focusing on these genes, the WGS revealed an average of 17,733 variants vs. 249.5 found with the SNP array. In silico analysis with the PROVEAN and SIFT algorithms, which are in silico functional predictors, showed some missense variants likely to be deleterious. Specifically, they found that 254 of the 332 variants in *UGT1A1* were novel, of which 31 were functional and 26 had a frequency of <1%. In general, the WGS approach allowed the identification of a significantly higher number of variants compared to the SNP array, which might impact the pharmacological processes.

Another study analysed sequencing data for 146 genes related to pharmacological traits from over 6500 individuals (data from the 1000 Genomes Project (1KGP) and Exome Sequencing Project (ESP)) (Table 5) [133]. They detected 19,328 single nucleotide variants (SNVs), 62.9% of which were exonic; for example, 6225 and 6258 variants in *ABC transporter* (22 genes) and *SLC* genes (49) respectively, and 253 variants in *UGTs* (16) and *GTSs* (14). Most of these variants were indeed rare (MAF < 1%; 92.9%) or very rare (MAF < 0.1%; 82.7%)—meaning that they would not be detected by conventional methods—and the majority were missenses (56.2%). The functional impact from rare variants was different across the genes, yet they concluded that rare variants contribute on average 30–40% of the functional variability in the studied pharmacogenes.

Schaller et al. analysed WES and WGS data from 141,456 individuals (data from gnomAD v2.1) and assessed the genetic variability of *SLC* genes (Table 5) [136]. They detected 204,287 SNVs and indels, of which 56.9% were missenses, and several were loss-of-function variants, such as 2.5% frameshifts, 1.7% stop-gains and 1.5% variations in canonical splice sites. They concluded that each individual presents, on average, 29.7 putatively functional *SLC* variants, with rare variants contributing 18% of this functional variability.

Following on from the results obtained from their initial GWAS, Rosmarin et al. sequenced the complete *DPYD* and *TYMS* coding regions and identified a further novel rare independent *DPYD* variant (c.1651G>A; p.Ala551Thr). This change was present in a single patient that had presented with grade 4 neutropenia and thrombocytopenia, and was predicted to be “strongly damaging” by in silico predictors (Table 4) [41].

NGS approaches can not only be useful to identify rarer variants but can be an important asset to reveal copy number variations (CNVs). The case in point is the work by Santos et al. that included CNV available data from 2504 whole genomes and 59,898 exomes (data from 1KGP and Exome Aggregation Consortium (ExAC)) and focused on 208 ADME genes (Table 5) [134]. Within these, 201 (97%) genes had a total of 5589 novel CNVs, where 47% were deletions and 54% were duplications. These novel deletions were responsible for >5% of loss-of-function alleles in a considerable number of genes (87, 25, 49, 48, 59 and 51 genes in non-Finnish Europeans, Finnish, East Asians, South Asians, Africans and admixed Americans, respectively). This demonstrates the impact that CNV might have on ADME genes, and hence the development of ADRs.

As the conventional screening methods only include common variants, a high number of variants are missed, thus explaining the need for unbiased and more comprehensive approaches. These interesting works emphasise the potential of NGS to detect novel rarer variants or CNV, not only in ADME genes, but in other pathways, which might help to explain the pharmacogenetic variability possibly associated with toxicity caused by chemotherapy.

## 5. Functional Assays

Functional assays on candidate variants are essential to ultimately clarify the mechanisms by which the genetic variants exert their effect on ADR development. Pharmacokinetic (PK) studies have been the most used approach to assess the functional impact of toxicity SNPs (Table 4). They have been used for many years now to evaluate enzymatic activity in patients carrying the desired variants, as they measure the level of drug and its metabolites that influence drug bioavailability and could hence lead to the toxicity profile.

By far, the most studied gene in PK studies has been *DPYD*, and there is an agreement that the DPD protein plays a crucial role in 5-FU metabolism. There are several methods to determine DPD deficiency [30,137]: testing for DPD activity in peripheral blood mononuclear cells, the uracil breath test, the uracil test dose and endogenous DHU/U ratio, or high-performance liquid chromatography (HPLC).

A study including 30 patients heterozygous for the *DPYD* rs3918290 variant analysed 5-FU plasma concentrations by HPLC and found that the mean maximum enzymatic 5-FU conversion capacity value was 40% lower in these patients (Table 6) [138].

Another study reported the effect of *DPYD* rs75017182 on DPD expression and activity and showed that heterozygous carriers presented a 35% activity reduction that was caused by alternative splicing [33].

By these means, at least four SNPs in *DPYD* have been proven deleterious: rs55886062, rs3918290, rs67376798 and rs56038477/HapB3 [30,34,156]. Studies on other variations have so far led to inconclusive or contradicting results [157].

Of late, other approaches have also been used to assess the functionality of pharmacogenetic variants. For instance, Offer et al. proposed the construction of a vector for rapid phenotypic assessment of *DPYD* variants and their relation with 5-FU sensitivity (Table 6) [29,31]. *DPYD* constructs were expressed in mammalian cells and the enzymatic activity of the expressed proteins was measured by HPLC and compared to the wild type. By these means, they could confirm that 30 of the variants caused a significant reduction in enzymatic activity. Interestingly, 19 of the variants tested displayed <25% activity. In turn, *DPYD* rs1801158, rs1801265, rs2297595, rs200687447, rs60139309 and rs114096998 had higher enzymatic activity, and therefore cells expressing these variants were more resistant to 5-FU, which may not confer susceptibility to toxicity development, but may in turn influence response rates.

In 2015, Henricks et al. proposed to assign an activity value (AV) to *DPYD* alleles, to adjust the initial dose of 5-FU. In this context, fully functional alleles had an AV = 1, reduced activity alleles had an AV = 0.5 and non-functional alleles had an AV = 0 (wild-type AV = 1; rs67376798 and rs56038477 AV = 0.5; and rs3918290 and rs55886062 AV = 0). Based on the AV of both alleles, the gene activity score (AS) is calculated, thus representing the enzymatic phenotype of the patient [30].

For genes other than *DPYD*, there is much less functional evidence (Table 6). Some research has been conducted on the relation of irinotecan PK variants. These studies were able to significantly associate polymorphisms in *ABCC1* and *ABCB1* with SN-38 exposure and the glucuronidation ratio (GR)—measured as AUC SN-38G/AUC SN-38 [73,111]. Demattia et al. investigated the possible association between *ABCG2* rs7699188 and *ABCB1* rs2032582 with irinotecan PK parameters on patients with advanced CRC by measuring plasma concentrations of irinotecan, SN38 and SN38G, but did not find any significant correlation [74]. Toffoli et al. evaluated irinotecan PK in 71 patients with metastatic CRC. They associated severe toxicity with a significantly lower GR (*p* = 0.01) and an increased biliary index (BI) (*p* = 0.003), which indicates SN-38 accumulation. Further, they reported a significant correlation between UGT1A1*28 and lower GR (*p* = 0.01), and higher BI (*p* = 0.007) [145]. Other works showed that patients with the wild-type genotype had a significantly higher clearance of SN-38 compared to UGT1A1*28 (*p* < 0.001), and that the homozygous genotype was significantly associated with GR (*p* = 0.005) and BI (*p* = 0.014) [151]. Iyer et al. also reported significantly lower SN-38 glucuronidation in patients with UGT1A1*28 (*p* = 0.001) [105]. Other *UGT1A* polymorphisms, such as UGT1A1*60 (*p* = 0.005), UGT1A1*93 (*p* < 0.0001), UGT1A1*6 (*p* = 0.037) and UGT1A7*3 (*p* < 0.02), were also associated with GR and BI [73,146,150].

## 6. Cost-Effectiveness Analysis

Besides the need for clear evidence on the functional relevance of a pharmacogenetic biomarker, a proof of cost-effectiveness—that the pharmacogenetic strategy is more effective with an acceptable additional cost or even a cost saving—is crucial to facilitate its introduction into clinical practice and acceptance from healthcare professionals and institutions.

In 2015, Deenen et al. evaluated the safety and costs of upfront DPYD*2A genotyping with individualised dose adjustment treatment for fluoropyrimidines [158]. They showed that genotype-guided dosing represented a reduction in severe toxicity from 73% to 28%. Moreover, dose adjustment based on genotype produced shorter and easier to control toxicities, and a significant reduction in drug-induced death from 10% to 0%. Therefore, they demonstrated that screening for DPYD*2A before treatment could be lifesaving and potentially cost-efficient. Cortejoso et al. complementarily evaluated the costs of genotyping three *DPYD* variants (rs3918290, rs67376798 and rs55886062) and the management of severe neutropenia caused by fluoropyrimidines. Considering an average cost of management of EUR 3044.40 vs. EUR 6.40 per patient for *DPYD* testing, they concluded that genotyping is cost-effective if severe neutropenia is prevented in at least 2.1 cases per 1000 treated patients [159]. Given that the combined frequency of these three markers is about 1%, this provides evidence that *DPYD* testing should be considered by healthcare systems. Murphy et al. further compared the reactive vs. prospective *DPYD* genotyping of variants rs3918290, rs67376798, rs1801158 and rs55886062. Of the 134 included patients, five carried a *DPYD* variant and the costs of their hospitalisation were EUR 232,061, whereas the total cost of genotyping prior to treatment for all patients would have been only EUR 23,718. Even if patients still had to endure some ADRs, the cost would have been considerably smaller, making pharmacogenetic analysis again cost-efficient [160]. In 2019, Henricks et al. also compared the costs from prospective *DPYD* screening (rs3918290, rs67376798, rs55886062 and rs56038477) with no screening on 1103 patients receiving fluoropyrimidine-based therapy. Patients with variants rs67376798 or rs56038477 had a 25% dose reduction, while patients with rs3918290 or rs55886062 had a 50% dose reduction. They concluded that the expected costs of the screening approach were EUR 2599 vs. EUR 2650 for the non-screening approach, representing a cost saving of EUR 51 per patient. These results strongly suggested that upfront *DPYD*-guided dose individualisation does not result in extra costs, and therefore solidly supports *DPYD* screening implementation prior to fluoropyrimidine treatment as a standard of care [161]. It also constituted the basis for pharmGKB EMA guideline changes from actionable to recommended.

Gold et al. assessed the safety and costs of testing for UGT1A1*28 before irinotecan treatment [162]. Assuming no treatment efficacy reduction, the average cost saving per patient was EUR 250. Obradovic et al. compared the standard irinotecan dose with dose reduction based on *UGT1A1* genotyping, and evaluated the cases of severe neutropenia, the number of life-years gained and the associated costs. They concluded that genotyping with dose reduction in homozygotes was cost-saving in African and Caucasian populations, but not in Asians, given the population frequency of this variant [163]. Another study by Butzke et al. compared severe neutropenia and grade 4 diarrhoea in a similar setting, to find that dosage calculations based on UGT1A1*28 genotypes save about EUR 600 per patient [164]. More recently, Roncato et al. calculated that the costs for toxicity management per patient increased 1.4-fold for heterozygotes and 6-fold for homozygotes compared to wild-type individuals, and they were superior to the costs related to genotyping all patients before treatment [165].

## 7. Pharmacogenomic Testing Guidelines

Although, as we have described so far, there is a considerable amount of evidence on the effect of genetic variants on CRC chemotoxicity, translation into clinical practice is yet far from routine implementation. For now, guidelines from leading authorities, including the European Medicines Agency (EMA), the Food and Drug Administration (FDA), the private pharmacogenetic consortia, the CPIC, the Royal Dutch Association for the Advancement of Pharmacy-Pharmacogenetics Working Group (DPWG) and the Spanish Agency for Medicines and Health Products (Agencia Española de Medicamentos y Productos Sanitarios, AEMPS) have only produced a very limited list of recommendations (Table 7) [166,167,168,169,170].

Pharmacogenetic guidelines from CPIC for the administration of fluoropyrimidines recommend that the *DPYD* metaboliser status (based on variants rs3918290, rs67376798, rs55886062 and rs56038477) is characterised prior to treatment administration. Poor metabolisers (AS 0–0.5) either: a) receive an alternative drug; or b) if 5-FU/capecitabine is still considered the better suited option of treatment, it is recommended to strongly reduce the given dose and accompany with close monitoring. For intermediate metabolisers (AS 1–1.5), a 50% dose reduction is recommended [156,167]. On the other hand, the FDA only contraindicates the administration of 5-FU/capecitabine in patients with DPD deficiency, but does not directly recommend screening for DPD deficiency before treatment, neither does it distinguish heterozygous nor homozygous DPD-deficient patients (www.pharmgkb.org/gene/PA145/labelAnnotation (accessed on 07 October 2020)) [170].

As for irinotecan treatments, pharmacogenomic testing criteria are merely based on the *UGT1A1* genotype (rs3064744). DPWG recommends a 30% reduction in the standard dose if patients are UGT1A1*28/28 [168], whereas the FDA vaguely recommends dose reduction (www.pharmgkb.org/chemical/PA450085/labelAnnotation (accessed on 07 October 2020)) [170]).

With the growing knowledge on CRC pharmacogenomics, more guidelines including other genes/variants will most likely be available in the next coming years. For instance, the *ABC* transporter genes, like *ABCC1* and *ABCB1*, have been quite studied so far and there is good evidence of their relation to the development of ADRs, both by association studies and functional assays.

## 8. Limitations in Pharmacogenomic Studies

In this appraisal, we have presented a comprehensive review of the field of CRC pharmacogenomics, since its early inception to the latest trends. Although remarkable findings have been produced, the road towards widespread clinical implementation is still far from over, and is inherently hindered by some of the limitations that pharmacogenetic analysis encounters. One of the main problems in pharmacogenomic studies is the extensive phenotype heterogeneity. This could be attributable to at least three different factors: (a) heterogeneity in clinical inclusion, i.e., differences in tumour staging and treatment strategies and lines (i.e., the genetic contribution to toxicity may be different in patients that have received FOLFOX as first-line treatment compared to those who have received it as second line); (b) pharmacogenomic data are not kept in a standardised manner, and it is usually hard to find in the patient’s clinical record case report forms, including the appropriate scaling, timing and line of treatment, and Eastern Cooperative Oncology Group (ECOG) performance status of the patient (amongst others) should therefore be used to produce robust study designs; and (c) some ADRs, like haematological counts, can be measured quantitatively, whereas others, like diarrhoea, are subject to clinician interpretation. To overcome this, toxicity grading scales such as the Common Terminology Criteria for Adverse Events (CTCAE) should be used across studies [172].

Secondly, the influence of each therapeutical agent alone is hard, if not impossible, to assess, as the great majority of patients undergo combination therapies, and many of the ADRs are shared amongst treatments. This could be due, for instance, to the backbone presence of 5-FU in most settings but could also result from a pleiotropic effect of different drugs.

Thirdly, there has been, in general, a lack of unambiguous association findings. This could be due to the abovementioned phenotypic heterogeneity, but also other factors such as study sample sizes or population stratification issues. For instance, the overwhelming majority of studies reported in this review have been performed exclusively on Caucasian populations, and there are few published works in non-Europeans. Moreover, those that have been published in Asians show considerable differences in the allelic frequencies of the variants. Therefore, validation of findings in cohorts with appropriate statistical power is essential. On this topic, an outstanding example is the Radiogenomics Consortium, which advocates for the standardisation of toxicity data collection derived from radiation treatments. They have published guidelines for STrengthening the Reporting Of Genetic Association studies in Radiogenomics (STROGAR), which allow for multi-institutional approaches towards large-scale radiotherapy patient biorepositories and databanks. Indeed, this consortium has already successfully completed several GWAS of radiotherapy toxicity [173,174,175,176,177].

Fourthly, there are no implemented guidelines for the reporting of pharmacogenetic studies. There have been recent efforts to overcome this, including a publication on the STrengthening the Reporting Of Pharmacogenetic Studies (STROPS). This work produces guidelines to standardise pharmacogenetic reporting. This could be essential for the homogenisation of pharmacogenetic data leading to improved systematic reviewing and meta-analyses, hence improving the power and applicability for pharmacogenetic associations [178].

Overall, the evidence gathered so far has brilliantly supported the relevance of pharmacogenomic testing in personalised medicine approaches. Novel genomic technologies such as GWAS and NGS offer unprecedented and affordable access to genomic information that can be assessed to discover novel pharmacogenomic variants related to toxic ADRs [179,180]. Pharmacokinetic profiling has proven to be useful for the identification of patients that might benefit from modified treatment strategies and might help improve the prediction value of genetic testing. Cost-effective analyses produced so far have validated the thought that the treatment design should be designed based on pharmacogenomic data, and that these strategies are always cost-effective vs. having to palliate toxicity issues.

Nevertheless, widespread testing is still anecdotic including in regulatory guideline recommendations. Researchers must hence make additional efforts to produce sound and relevant data that can be presented to the regulatory agencies to support pre-treatment testing. Surely, we must continue working in this direction towards a more meaningful implementation of pharmacogenomics in the routine clinical practice.

## Figures and Tables

**Table 1:** Guidelines for colorectal cancer (CRC) treatment.

CRC Stage	Treatment	
	Surgery	Pharmacological Treatment
I	Wide surgical resection and anastomosis	No adjuvant chemotherapy recommended
II	Wide surgical resection and anastomosis	Adjuvant chemotherapy for high-risk could be considered
III	Wide surgical resection and anastomosis	Adjuvant administration of oxaliplatin plus 5-FU or capecitabine
IV	The majority of patients have metastases that initially are not suitable for potentially curative resection. Revaluate after chemotherapy	Cytotoxic agents: 1st line: 5-FU or capecitabine alone or in combination either with oxaliplatin or irinotecan 2nd line: if refractory to irinotecan-based treatment, FOLFOX is recommended; and if refractory to oxaliplatin-based treatment, FOLFIRI is recommended
		Biological targeted agents: 1st line: monoclonal antibodies against VEGF (bevacizumab, aflibercept) and/or EGFR (cetuximab, panitumumab), if *RAS* mutation excluded Multi-kinase inhibitor: regorafenib

FOLFOX: folinic acid (leucovorin-LV) + fluorouracil + oxaliplatin; FOLFIRI: leucovorin + fluorouracil + irinotecan; VEGF: vascular endothelial growth factor; EGFR: epidermal growth factor receptor.

**Table 2:** The most common toxicity profile of CRC treatments.

Treatment	Significant ADRs (According to FDA Labels) *	ADR Incidence (% Patients)	Ref.
5-Fluororacil	Diarrhoea, neutropenia, mucositis, nausea/vomiting, stomatitis, asthenia, leukopenia, anaemia.	94%	[12]
Capecitabine	Hand-and-foot syndrome, diarrhoea, nausea/vomiting, abdominal pain, fatigue, hyperbilirubinemia.	96%	[12]
Oxaliplatin	Peripheral sensory neuropathy, neutropenia, thrombocytopenia, anaemia, nausea/vomiting, increase in transaminases and alkaline phosphatase, diarrhoea, fatigue, stomatitis.	>92%	[15]
Irinotecan	Nausea/vomiting, diarrhoea, neutropenia, alopecia, abdominal pain, constipation, anorexia, leukopenia, anaemia, asthenia, fever, body weight decreasing.	100%	[16]
Cetuximab	Cutaneous adverse reactions, headache, diarrhoea, infection.	>87%	[17]
Panitumumab	Skin rash, paronychia, fatigue, nausea, diarrhoea.	>90%	[18]
Bevacizumab	Haemorrhage, hypertension, headache, rhinitis, proteinuria, taste alteration, dry skin, lacrimation disorder, back pain, exfoliative dermatitis.	>60%	[19]

* According to Food and Drug Administration (FDA) label section: Warnings and Precautions, Contraindications, and Boxed Warning Sections of Labelling for Human Prescription Drug and Biological Products.

**Table 3:** Summary of CRC pharmacogenomics.

Drug	Gene	SNP (rsID)	Change	Alternative Nomenclature	Frequency of Risk Allele ^a^	Associated ADR	OR (95% CI)	Evidence Level ^b^	Ref.
Fluoropyrimidines	*DPYD*	rs55886062	NM_000110.3:c.1679T>G; NP_000101.2:p.Ile560Ser	DPYD*13	3 × 10^−4^ (C)	Global toxicity	6.0 (0.6–61)	1A	[28]
		rs3918290	NM_000110.4:c.1905+1G>A (Splice donor)	DPYD*2A	0.007 (T)	Global toxicity	8.5 (1.8–40.9)	1A	[29]
		rs67376798	NM_000110.3:c.2846A>T; NP_000101.2:p.Asp949Val		0.003 (A)	Global toxicity	7.8 (1.6–39.2)	1A	[31]
		rs115232898	NM_000110.3:c.557A>G; NP_000101.2:p.Tyr186Cys		0.002 (Afr: 0.023) (C)	Neutropenia, mucositis, alopecia	-	1A	[32]
		rs75017182	NM_000110.4:c.1129-5923C>G (Intronic)		0.013 (C)	Global toxicity	6.8 (2.0–23)	1A	[33]
		rs56038477	NM_000110.3:c.1236G>A; NP_000101.2:p.Glu412=		0.014 (T)	Gastrointestinal; haematological	2.0 (1.5–2.8) 2.8 (1.2–3.7)	3	[34]
		rs72549303 c	NM_000110.4:c.1898del; NP_000101.2:p.Pro633fs	DPYD*3	NA	NA	NA	1A	[31]
		rs72549309 c	NM_000110.4:c.295_298TCAT [1]; NP_000101.2:p.Phe100fs	DPYD*7	6 × 10^−5^ (delATGA)	NA	NA	1A	[31]
		rs1801266 c	NM_000110.4:c.703C>T; NP_000101.2:p.Arg235Trp	DPYD*8	3 × 10^−5^ (A)	NA	NA	1A	[31]
		rs1801268 c	NM_000110.4:c.2983G>T; NP_000101.2:p.Val995Phe	DPYD*10	NA	NA	NA	1A	[31]
		rs78060119	NM_000110.3:c.1156G>T; NP_000101.2:p.Glu386Ter	DPYD*12	8 × 10^−6^ (A)	Leucopenia, thrombocytopenia, mucositis	NA	1A	[35]
		rs2297595	NM_000110.3:c.496A>G; NP_000101.2:p.Met166Val		0.085(C)	Global toxicity	5.9 (1.3–27.2)	3	[36]
		rs1801265	NM_000110.3:c.85T>C; NP_000101.2:p.Cys29Arg	DPYD*9A	0.228 (G)	Diarrhoea	0.8 (0.7–1)	3	[37]
		rs1801267 c	NM_000110.4:c.2657G>A; NP_000101.2:p.Arg886His	DPYD*9B	1 × 10^−4^ (T)	NA	NA	NA	[38]
		rs1801159	NM_000110.3:c.1627A>G; NP_000101.2:p.Ile543Val	DPYD*5	0.198 (C)	Diarrhoea	4.9 (-)	3	[39]
		rs1801158	NM_000110.3:c.1601G>A; NP_000101.2:p.Ser534Asn	DPYD*4	0.015 (T)	Global toxicity	1.7 (1.1–2.6)	3	[37]
		rs17376848	NM_000110.3:c.1896T>C; NP_000101.2:p.Phe632=		0.051 (G)	Global toxicity	14.5 (1.4–155.2)	3	[36]
		rs1801160	NM_000110.3:c.2194G>A; NP_000101.2:p.Val732Ile	DPYD*6	0.048 (T)	Global toxicity	2.1 (1.5–3.0)	3	[40]
		rs12022243	NM_000110.4:c.1906-14763G>A (Intronic)		0.181 (T)	Global toxicity	1.7 (1.5- 1.9)	3	[41]
		rs12119882	NM_000110.4:c.680+2545T>C (Intronic)		0.075 (G)	Hyperbilirubinemia	4.9 (1.2–20.8)	3	[42]
		rs76387818	Intergenic		0.019 (A)	Global toxicity	4.1 (3.5–4.6)	3	[41]
		rs12132152	Intergenic		0.020 (A)	HFS;global toxicity	6.1 (5.5–6.8); 1.6 (1.4–1.8)	3	[41]
	*TYMS*	rs183205964	NM_001071.4:c.-86= (5′ UTR)		3 × 10^−5^ (C)	Global toxicity	3.0 (1.1- 8.4)	3	[43]
		rs2853741	NM_001071.4:c. (Upstream transcript)		0.322 (T)	Diarrhoea	0.3 (0.1–0.7)	3	[42]
		rs699517	NM_017512.7:c.*1289= (3′ UTR)		0.379 (T)	Nausea/vomiting;asthenia	7.9 (1.5–41.6); 0.3 (0.1–0.8)	3	[42]
		rs45445694	NM_001071.4:c. (5′ UTR)		0.007 (2R2R)	Global toxicity	1.7 (-)	3	[44]
		rs2853542	NM_001071.4:c.-58= (5′ UTR)			Global toxicity; HFS	1.5 (1.2–1.8); 1.4 (1.2–1.8)	NA	[45]
		rs11280056	NM_017512.7:c.*853_*861= (3′ UTR)			Global toxicity	1.7 (1.2–2.2)	NA	[45]
	*ENOSF1*	rs2612091	NM_017512.7:c.742-227G>C (Intronic)		0.373 (C)	Global toxicity	1.6 (1.4–1.8)	3	[41]
	*UMPS*	rs2279199	NM_000373.4:c. (Genic upstream transcript)		0.556 (T)	Nausea/vomiting	0.2 (0.1–1.0)	3	[42]
		rs4678145	NM_000373.4:c.156+607G>C (Intronic)		0.096 (C)	Asthenia	4.5 (1.6–13.2)	3	[42]
		rs1801019 d	NM_000373.4:c.638G>C; NP_000364.1:p.Gly213Ala		0.169 (C)	Global toxicity	17.6 (1.6–195.9)	3	[46]
	*MTHFR*	rs1801131	NM_001330358.1:c.1409A>C; NP_001317287.1:p.Glu470Ala		0.289 (G)	HFS	10.0 (3.8–27.8)	3	[47]
		rs1801133	NM_001330358.1:c.788C>T; NP_001317287.1:p.Ala263Val		0.315 (A)	Neutropenia	2.3 (1.2–4.6)	3	[48]
	*TYMP*	rs11479	NM_001113755.3:c.1412C>T; NP_001244917.1:p.Ser471Leu		0.094 (A)	Global toxicity	2.7 (1.2–5.9)	3	[49]
	MIR27A	rs895819	NR_029501.1:n.40A>G (Non-coding transcript)		0.335 (C)	Global toxicity	1.6 (1.1–2.2)	3	[50]
	*ABCC1*	rs7194667	NM_032583.4:c.1609-491A>C (Intronic)		0.063 (G)	Leucopenia	3.31 (1.3–8.7)	3	[51]
	*ABCB1*	rs1045642	NM_001348945.1:c.3645T>C; NP_001335874.1:p.Ile1215=	ABCB1*6	0.504 (G)	HFS	NA	3	[52]
		rs2032582	NM_001348945.1:c.2887T>G; NP_001335874.1:p.Ser963Ala	ABCB1*7	0.637 (C)	HFS	NA	3	[52]
		rs1128503	NM_001348945.1:c.1446T>C; NP_001335874.1:p.Gly482=	ABCB1*8	0.614 (G)	Neutropenia	NA	3	[52]
	*SLC22A7*	rs2270860	NM_006672.3:c.1269C>T; NP_006663.2:p.Ser423=		0.368 (T)	Global toxicity	17.1 (1.7–170.3)	3	[42]
		rs4149178	NM_006672.3:c.1586+206A>G (Intronic)		0.795 (A)	Diarrhoea	0.3 (0.1–0.9)	3	[42]
	*CDA*	rs2072671	NM_001785.3:c.79A>C; NP_001776.1:p.Lys27Gln		0.279 (C)	Global toxicity	1.8 (1.1–3.0)	3	[53]
		rs1048977	NM_001785.3: c.435C>T; NP_001776.1:p.Thr145=		0.307 (T)	Hyperbilirubinemia	8.6 (1.1–70.3)	3	[42]
		rs602950	NM_001785.3:c. (Upstream transcript)		0.224 (G)	Diarrhoea	2.3 (1.3–4.2)	3	[47]
		rs3215400	NM_001785.3:c.-33_-31= (5′ UTR)		0.555 (delC)	HFS	0.5 (0.3–1.0)	3	[54]
		rs532545	NM_001785.3:c. (Upstream transcript)		0.220 (T)	Diarrhoea	2.3 (1.3–4.2)	NA	[47]
	*CES1*	rs3217164	NM_001025195.2:c.693+129del (Intronic)		0.607 (G)	Global toxicity	4.1 (1.8–9.0)	3	[55]
		rs2244614	NM_001025195.2:c.1171-41C>T (Intronic)		0.482 (G)	Global toxicity	4.7 (1.9–12.0)	3	[55]
		rs2244613	NM_001025195.2:c.1171-33C>T (Intronic)		0.232 (G)	Global toxicity	6.4 (1.5–27.7)	3	[55]
	*CES1P1*	rs7187684	NR_003276.2:n. (Intronic)		0.278 (T)	Global toxicity	6.5 (1.5–28.0)	3	[55]
		rs11861118	NR_003276.2:n. (Upstream transcript)		0.161 (G)	Global toxicity	6.5 (1.5–28.0)	3	[55]
	Intergenic	rs9936750	Intergenic		0.161 C	Global toxicity	4.6 (1.5–13.9)	3	[56]
	Intergenic	rs10876844	Intergenic		0.439 (A)	Diarrhoea	6.5 (1.6–27.2)	NA	[57]
Oxaliplatin	*ABCC2*	rs717620	NM_000392.5:c.-24= (5‘ UTR)		0.171 (T)	Neuropathy	14.4 (1.6–127.0)	3	[58]
		rs3740066	NM_000392.5:c.3972C>TNP_000383.2:p.Ile1324=			Neuropathy	3.0 (1.2–7.7)	NA	[58]
		rs1885301	NM_000392.5:c. (Upstream Transcript)		0.413 (A)	Neuropathy	3.1 (1.4–6.9)	NA	[58]
		rs4148396	NM_000392.5:c.3258+56T>C (Intronic)		0.347 (T)	Neuropathy	4.7 (1.6–13.7)	NA	[58]
	*ABCG2*	rs3114018	NM_004827.3:c.-19-3415T>G (Intronic)		0.516 (A)	Neuropathy	2.7 (1.0–4.4)	NA	[59]
	*GSTP1*	rs1695	NM_000852.3:c.313A>G; NP_000843.1:p.Ile105Val	GSTP1*B	0.339 (G)	Dying	3.0 (1.2–7.6)	3	[60]
	*GSTM1*	Null genotype	-	GSTM1*0		Neutropenia	2.0 (1.1–3.7)	NA	[61]
	*GSTT1*	Null genotype	-			Neutropenia	2.0 (1.1–3.7)	NA	[61]
	*ERCC1*	rs11615	NM_202001.3:c.354T>C; NP_001356337.1:p.Asn118=		0.498 (A)	Neutropenia	4.6 (1.2–17.4)	3	[48]
	*ERCC2*	rs13181	NM_000400.3:c.2251A>C; NP_000391.1:p.Lys751Gln		0.323 (G)	Haematological	2.2 (1.3–3.8)	3	[62]
		rs238406	NM_000400.4:c.468A>C NP_000391.1:p.Arg156=		0.645 (C)	Thrombocytopenia	NA	NA	[63]
	*PARD3B*	rs17626122	NM_001302769.2:c.3261-6168T>C (Intronic)		0.550 (T)	Global toxicity	3.4 (1.9–6.8)	3	[57]
	Intergenic	rs7325568	Intergenic		0.409 (T)	Haematological	1.8 (1.3–2.4)	3	[57]
Irinotecan	*UGT1A1*	rs3064744	NM_000463.3:c.(Upstream transcript)	UGT1A1*28	0.347 (dupTA) (EAS:0.122)	Global toxicity	7.2 (2.5–22.3)	2A	[64]
		rs4148323 c	NM_000463.2:c.211G>A; NP_000454.1:p.Gly71Arg	UGT1A1*6	0.014 (EAS: 0.144) (A)	NA	NA	2A	[65]
		rs11563250	NM_001367507.1:c. (Genic upstream transcript)		0.893 (A)	Neutropenia	0.3 (0.2–0.6)	3	[66]
		rs4124874	NM_001072.3:c.862-10021T>G (Intronic)	UGT1A1*60	0.452 (T)	Neutropenia	NA	3	[67]
		rs10929302	NM_019075.2:c.856-9898G>A (Intronic)	UGT1A1*93	0.299 (A)	Global toxicity	8.4 (1.9–37.2)	3	[68]
	*UGT1A9*	rs11692021	NM_021027.3:c.855+9770T>C (Intronic)		0.349 (C)	Global toxicity	2.0 (1.1–3.6)	3	[69]
		rs3832043 e	NM_021027.3:c. (Upstream Transcript)		0.609 (delT)	Diarrhoea	6.3 (1.3–31.7)	3	[70]
	*UGT1A6*	rs2070959	NM_001072.4:c.541A>G (Intronic)		0.689 (A)	Global toxicity	2.1 (1.1–3.9)	3	[69]
	*ABCG1*	rs225440	NM_016818.3:c.286+7029C>T (Intronic)		0.428 (T)	Neutropenia	3.1 (1.1–8.6)	3	[71]
		rs425215	NM_016818.3:c.974-898C>G (Intronic)		0.623 (G)	Gastrointestinal	11.4 (1.7–78.4)	NA	[72]
	*ABCB1*	rs12720066	NM_001348945.1:c.2529+971T>G (Intronic)		0.035 (C)	Neutropenia	NA	3	[73]
	*ABCC1*	rs17501331	NM_004996.4:c.49-12232A>G (Intronic)		0.928 (A)	Neutropenia	NA	3	[73]
		rs3743527	NM_004996.4:c.*543= (3′ UTR)		0.774 (C)	Neutropenia	NA	3	[73]
	*ABCC5*	rs2292997	NM_005688.4:c.129+7980C>T (Intronic)		0.126 (A)	Neutropenia	3.2 (1.3–7.9)	3	[71]
		rs10937158	NM_005688.4:c.130-1268A>T (Intronic)		0.612 (C)	Diarrhoea	0.4 (0.2–0.8)	3	[71]
		rs3749438	NM_005688.4:c.591+374C>T (Intronic)		0.324 (A)	Diarrhoea	5.9 (1.3–26.3)	3	[71]
		rs562	NM_005688.4:c.*1243= (3′ UTR)		0.515 (C)	Gastrointestinal	32.0 (2.8–370.8	NA	[72]
	*ABCG2*	rs7699188	NM_004827.3:c. (Genic upstream transcript)		0.227 (A)	Global toxicity; non-haematological	7.3 (1.5–34.5); 15.2 (2.5–78.2)	3	[74]
	*SLCO1B1*	rs2306283	NM_006446.5:c.388A>G NP_006437.3:p.Asn130Asp	SLCO1B1*1b	0.529 (G)	Gastrointestinal	2.3 (0.4–15.1)	NA	[72]
	*TOP1*	rs6072262	NM_003286.4:c.279+61G>A (Intronic)		0.144 (A)	Neutropenia	NA	3	[75]
	*TGFBR2*	rs3087465	NM_001024847.2:c. (2KB upstream)		0.659 (G)	Diarrhoea	3.7 (1.0–13.3)	3	[76]
	*TGFB1*	rs1800469	NM_000660.7:c. (Upstream transcript)		0.701 (G)	Diarrhoea	4.4 (1.0–18.9)	3	[76]
	*KCNQ5*	rs9351963	NM_019842.4:c.490-1798A>C (Intronic)		0.178 (C)	Diarrhoea	3.3 (1.8–5.6)	3	[77]
	Intergenic	rs10486003	Intergenic		0.913 (C)	Neuropathy	0.3 (0.2–0.5)	NA	[78]
	Intergenic	rs2338	Intergenic		0.275 (A)	Neuropathy	2.3 (1.6–3.3)	NA	[78]
	Intergenic	rs830884	Intergenic		0.92 (T)	Neuropathy	0.3 (0.2–0.5)	NA	[78]
	*ACYP2*	rs843748	NM_001320586.2:c.405-28913G>A (Intronic)		0.379 (A)	Neuropathy	2.4 (1.6–3.7)	NA	[78]
	*DLEU7*	rs797519	NC_000013.11:g.50656996G>C (Intronic)		0.548 (G)	Neuropathy	0.5 (045–0.7)	NA	[78]
	*FARS2*	rs17140129	NM_001318872.2:c.-22+36771A>G (Intronic)		0.158 (G)	Neuropathy	3.3 (1.8–6.4)	NA	[78]
Cetuximab	*EGFR*	rs712830	NM_005228.5:c.-191= (5′ UTR)		0.894 (C)	Global toxicity	6.1 (1.6–23.8)	3	[79]
		rs2227983	NM_005228.5:c.1562G>A NP_005219.2:p.Arg521Lys		0.768 (G)	Skin toxicity	3.2 (1.3–8.3)	3	[80]
		rs11568315	NM_005228.5:c.88+1195AC [10] (Intronic)		3.9 × 10^−4^ (CA > 35)	Skin toxicity	2.9 (1.0–8.9)	NA	[81]
	*RPS7*	rs10203413	NC_000002.12:g.3581588G>A (Regulatory region)		0.776 (G)	Skin toxicity	0.1 (0.1–0.4)	NA	[82]
	*ZNF827*	rs12646351	NC_000002.12:g.3581588G>A (Intronic)		0.815 (G)	Skin toxicity	0.04 (0.01–0.3)	NA	[82]
		rs17806780	NM_001306215.2:c.2383+11920A>T (Intronic)		0.818 (T)	Skin toxicity	0.04 (0.01–0.4)	NA	[82]
	*EPHA5*	rs7692430	NM_004439.8:c.2237-1876A>G (Intronic)		0.156 (G)	Skin toxicity	4.6 (2.5–8.5)	NA	[82]
Bevacizumab	*VEGF*	rs3025039	NM_001171623.1:c.*237= (3′ UTR)		0.134 (T)	Hypertension	0.2 (0.03–0.8)	NA	[83]
		rs2010963	NM_001171623.1:c.-634= (5′ UTR)		0.698 (G)	Hypertension	NA	NA	[84]
		rs833061	NM_001025366.3:c. (Upstream transcript)		0.452 (C)	Hypertension	0.2 (0.03–0.8)	NA	[85]
		rs699947	NM_001025366.3:c. (Upstream transcript)		0.414 (A)	Hypertension	0.1 (0.01–0.6)	NA	[85]

a: The risk alleles frequencies were consulted on gnomAD. b: Measure of confidence in the association, according to PharmGKB [10]. c: Associated with changes in enzymatic activity, but with a particular adverse drug reaction (ADR). d: Described for tegafur, a prodrug of 5-FU. e: Described for non-small-cell lung carcinoma. NA: not available. Note: In case of multiple studies, we have chosen a publication used by PharmGKB to support the level of evidence of the referred variant, and the corresponding OR and *p*-value.

**Table 4:** Advantages and disadvantages of different pharmacogenomics approaches.

Approach	Advantages	Disadvantages
Candidate genes	offers biological plausibility associates variants with known functional consequences and direct clinical implication	bias toward certain genes/pathways (usually, ADME genes) based on prior information of relevance to phenotype, which may be incomplete unable to discover novel genes/pathways the selected SNPs may not represent the full variation of the studied genes limited to protein-coding regions
SNP arrays (GWAS)	unbiased by a priori functional knowledge potential discovery of other relevant genes/pathways potential to identify variation in regulatory regions such as promoters or enhancers high-throughput	need to be adequately powered to detect moderate-effect variants require large sample sizes multiple testing correction needs to be applied variants might be intergenic; harder to interpret inspects common populational variation (potential loss of rarer variants) not suitable for CNV studies
SNP arrays (targeted fine-mapping approaches)	denser coverage cheaper may be population-specific	design bias may require a priori knowledge of region to study (i.e., as defined by GWAS, for example).
NGS (targeted panels, WES, WGS)	possibility of densely resequencing an entire gene (targeted genes) allows a more comprehensive and unbiased identification of novel genetic biomarkers allows the identification of relevant rare variants and CNV rapid evolution of NGS technologies	large number of false positives and VUS need for validation by Sanger or other genotyping methods higher turnaround time and costs (although decreasing) need for high data storage capacity need for deeper bioinformatic knowledge
Functional assays	give mechanistic perspective on how variants exert their effect validate the findings at the molecular level, giving further validity to the statistical association results potentially applicable to a specific desired tissue	assay design may be difficult, particularly in the case of intergenic variants results must be replicated in clinical studies

**Table 5:** Summary of relevant next-generation sequencing (NGS) results.

N	Cohort	Method	Genes	Results	Ref.
482	Genomes Data, Wellderly Study	WGS or SNP array genotyping	231 pharmacogenes	≈17,733 (WGS) vs. 249.5 (SNP array) *UGT1A1* (WGS): 254 of 332 variants were novel, 31 functional and 26 with frequency < 1%.	[131]
>6500	1KG phase 3; ESP	WES and WGS	146 pharmacogenes	19,328 SNV, 62.9% exonic 6225 and 6258 variants in *ABC* transporter (22 genes) and SLC genes (49), respectively, 253 variants in *UGTs* (16) and *GTSs* (14) 92.9% rare, 82.7% very rare 56.2% missenses ≈30–40% of the functional variability in pharmacogenes	[133]
141,456	gnomAD v2.1 ^a^	WES and WGS	*SLC* genes	204,287 SNVs and indels, 56.9% missenses, 2.5% frameshifts, 1.7% stop-gains and 1.5% variations in canonical splice sites Each individual had ≈29.7 putatively functional *SLC* variants, 18% of functional variability due to rare variants	[136]
100	QUASAR	Amplicon sequencing	*DPYD* and *TYMS* coding regions	Novel rare independent *DPYD* variant (c.1651G>A; p.Ala551Thr)—classified as strongly damaging	[41]
62,402	1 KG phase 3; ExAC ^b^	WES and WGS	208 pharmacogenes	201 (97%) genes had 5589 novel CNVs, 47% deletions and 54% duplications Novel deletions responsible for >5% of loss-of-function alleles in 87, 25, 49, 48, 59 and 51 genes in non-Finnish Europeans, Finnish, East Asians, South Asians, Africans and admixed Americans, respectively	[134]

1 KG: 1000 Genomes Project; ESP: Exome Sequencing Project; a: non-Finnish Europeans, Finns, Africans, East Asians, South Asians, Latinos, Ashkenazi Jews and other populations; ExAC: Exome Aggregation Consortium; b: included six major populations: non-Finnish Europeans, Finns, Africans, South Asians, East Asians and admixed Americans.

**Table 6:** Pharmacokinetic studies on fluoropyrimidines and irinotecan.

Genes	Significant Variants	N	Pharmacokinetic Results	Ref.
Fluoropyrimidines				
*DPYD*	rs3918290	1 case (heterozygous for IVS14+1G>A) vs. 6 controls (CRC)	inactivation of one *DPYD* allele: strong ↓CL_5-FU_: severe toxicity	[139]
*DPYD*	rs1801265 rs115232898 rs55886062	175 CRC patients	rs55886062: lowest activity (*p* = 0.014) rs115232898: 46% ↓activity (*p* = 0.026) rs1801265: 27% ↑activity (*p* = 0.013)	[140]
*DPYD*	rs3918290 rs67376798 rs55886062	487 advanced carcinoma patients	rs3918290, rs67376798, or rs55886062: ↓CL_5-FU_ (*p* < 0.001)	[141]
*DPYD*	rs3918290	30 patients (heterozygous for IVS14+1G>A) and 18 controls	rs3918290: 40% ↓Vmax (*p* < 0.001)	[142]
*DPYD*	rs1801159	112 gastric or colon cancer patients	rs1801159: ↓k (*p* = 0.022) and nausea/vomiting (*p* = 0.005)	[143]
*DPYD*	rs55886062 rs1801265 rs1801158	Expression vector	rs1801158: 36% ↑activity (*p* = 3.4 × 10^−7^) rs1801265: 13% ↑activity (*p* = 0.0013) rs55886062: 75% ↓activity (*p* = 5.2 × 10^−9^)	[29]
*DPYD*	rs141044036 rs72549308 rs1801268 rs145773863 rs55674432 rs72547601 rs137999090 rs59086055 rs1801266 rs111858276 rs183385770 rs72549307 rs138616379 rs72549304 rs112766203 rs183105782 rs143986398 rs115232898 rs2297595	Expression vector	rs141044036, rs72549308, rs1801268, rs145773863, rs55674432, rs137999090, rs72547601, rs59086055: <12.5% activity (*p* < 3.5 × 10^−4^) rs1801266, rs72549307, rs111858276, rs138616379, rs183385770, rs72549304: 12.5–25% activity (*p* < 0.0021) rs112766203, rs143986398, rs183105782, rs115232898: >25% ↓activity (*p* < 0.05) rs2297595: 120% ↑activity (*p* = 0.025)	[31]
*ABC*	rs2271862	48 CRC patients	*ABCA2* rs2271862: ↑CL_5-FU_	[144]
*ABCB1* *ABCC1* *ABCG2* *UGT1A1*	rs12720066 rs6498588 rs10929302	85 advanced cancer patients	*ABCB1* rs12720066 (*p* = 6.24 × 10^−4^) and rs6498588 (*p* = 9.50 × 10^−4^), and *UGT1A1* rs10929302 (*p* = 9.00 × 10^−5^): ↑AUC_SN-38_ ↑AUC_SN-38_: G ≥ 3 neutropenia (*p* = 0.0001)	[73]
*ABCG2* *SLCO1B1* *ABCB1* *ABCC1* *ABCC2* *UGT1A1* *UGT1A9* *UGT1A7* *CES* *CYP3A4* *CYP3A5* *HNF1A*	rs717620 rs1169288 rs4149056 rs35605 rs1092302 rs3740066	85 advanced cancer patients	*ABCC2* rs717620 (*p* = 0.002), *HNF1A* rs1169288 (*p* = 0.007), *SLCO1B1* rs4149056 (*p* = 0.015): ↑AUC_CPT-11_ *ABCC1* rs35605 (*p* = 0.031), UGT1A1 rs1092302 (*p* = 0.007): ↑AUC_SN-38_ *ABCC2* rs3740066: ↑AUC_SN-38G_ and ↑AUC_APC_ (*p* = 0.012) *ABCC1* rs35605 (*p* = 0.023), rs3064744 (*p* < 0.0001): ↓GR	[111]
*UGT1A1*	rs3064744	250 mCRC patients	↓GR (*p* = 0.01) and ↑BI (*p* = 0.003): G ≥ 3 toxicity rs3064744: ↓GR (*p* = 0.01) and ↑BI (*p* = 0.007)	[145]
*UGT1A1* *UGT1A7* *UGT1A9*	rs4124874 rs10929302 UGT1A7*3	Subset of 71 patients	*UGT1A1* rs4124874 and rs10929302: ↑BI (*p* = 0.03 and *p* = 0.04, respectively) UGT1A7*3: ↓GR (*p* = 0.02) and ↑BI (*p* = 0.007)	[146]
*HNF1A*	rs2244608	Subset of 49 patients	rs2244608: ↑AUC_SN-38_ (*p* = 0.032), ↑BI (*p* = 0.021) and ↓GR (*p* = 0.035)	[147]
*ABCC2*	rs2273697 rs17216114 rs1885301 rs2804402 rs717620 rs3740066	31 mCRC patients	rs2273697: ↓AUC_CPT-11_ (*p* = 0.011) rs17216114: ↓AUC_SN-38_ rs1885301, rs2804402, rs717620 and rs3740066: ↑AUN_SN-38_ (*p* < 0.03)	[148]
*ABCB1* *ABCC1* *ABCC2* *ABCG2* *CES1* *CES2* *CYP3A4* *CYP3A5* *UGT1A* *XRCC1*	rs1128503	65 solid tumour patients	*ABCB1* rs1128503: ↑AUC_CPT-11_ (*p* = 0.038), AUC_SN-38_ (*p* = 0.031) and ↓CL_SN-38_ (*p* = 0.015)	[96]
*UGT1A1*	rs3064744	20 solid tumour patients	rs3064744 ↓GR (*p* = 0.001) and ↑BI (*p* = 0.001) AUC_SN-38_: neutropenia (*p* < 0.0001)	[105]
*UGT1A1* *UGT1A9*	rs3064744	94 solid tumour patients	rs3064744: ↓GR (*p* = 0.022)	[149]
*UGT1A1*	rs4148323 rs4124874 rs3064744	85 solid tumour patients	rs4148323: ↓GR (*p* = 0.0372) rs4124874: ↑BI (*p* = 0.0048) rs3064744: ↑BI (*p* = 0.0007)	[150]
*ABCC2* *UGT1A1*	ABCC2*2 rs306474	167 solid tumour patients	ABCC2*2: ↓CL_CPT-11_ (*p* = 0.020) rs3064744: ↓CL_SN-38_ (*p* < 0.001), GR and BI (*p* = 0.014)	[151]
*UGT1A1*	rs3064744	62 solid tumour patients	rs3064744: ↓CL_SN-38_ (*p* < 0.01) ↑SN-38 exposure: G2–3 diarrhoea (*p* = 0.03)	[152]
*UGT1A1*	rs3064744	65 solid tumour or lymphoma patients	rs3064744: ↑BI (*p* = 0.0003) and ↓GR (*p* = 0.03) ↑BI: G4 neutropenia (*p* = 0.001)	[67]
*UGT1A1* *UGT1A7* *UGT1A9* *UGT1A10*	rs3064744 rs4148323	176 cancer patients	rs3064744 or rs4148323: ↓GR (*p* < 0.0001)	[153]
*UGT1A1* *ABCG2*	rs4148323	45 cancer patients	rs4148323: ↑AUC_SN-38_ (*p* = 0.018), ↓GR (*p* = 0.006) and 61% ↑BI (*p* = 0.003)	[154]
*ABCB1*	ABCB1*2	49 cancer patients	ABCB1*2: ↓CL_CPT-11_, _SN-38_, _APC_ (*p* = 0.0154, 0.0043, 0.0169, respectively)	[155]

Enzymatic activities were measured by high performance liquid chromatography (HPLC).

**Table 7:** Current CRC pharmacogenetic guidelines for treatment administration.

Drug	Gene	Annotation by Drug Regulatory Agencies and Guidelines Recommendations				
		FDA	CPIC	AEMPS	EMA	DPWG
Fluoropyrimidines	*DPYD*	Actionable PGx ^a^ Withhold or permanently discontinue treatment in patients with evidence of acute early-onset or severe toxicity, which may indicate near complete or absence of DPD activity. No dose has been proven safe for patients with no DPD activity. There is insufficient data to recommend a specific dose in patients with partial DPD activity [170]	Actionable PGx ^a^ Intermediate metaboliser (individual with one normal function allele plus one no function allele or one decreased function allele, or with two decreased function alleles)—decreased DPD activity and increased risk for severe/fatal ADR. Reduce starting dose based on AS followed by titration of dose based on toxicity or therapeutic drug monitoring (if available). AS 1: Reduce dose by 50% AS 1.5: Reduce dose by 25–50% Poor metaboliser (individual with two no function alleles, or with one no function plus one decreased function allele)—complete DPD deficiency and increased risk for severe/fatal ADR. AS 0.5: Avoid treatment, and in case alternative agents are not suitable, strongly reduce starting dose with early therapeutic drug monitoring. AS 0: Avoid treatment [167]	Testing required ^b^ Test for *DPYD* genotype (c.1905+1G>A, c.1679T>G, c.2846A>T and c.1236G>A/HapB3) and/or DPD deficiency (measure blood uracil level) before treatment. Treatment is contraindicated in patients with complete DPD deficiency. In case of partial DPD deficiency with no suitable alternative agents, reduce initial dose and monitor levels. No concrete reduction has been established [166]	Testing required ^b^ Test for the lack of DPD activity before treatment (measure blood uracil level, or check for *DPYD* variants—c.1905+1G>A, c.1679T>G, c.2846A>T y c.1236G>A/HapB3). Treatment is contraindicated in patients with complete DPD deficiency. A reduced starting dose should be considered in patients with partial DPD deficiency [169]	-
Irinotecan	*UGT1A1*	Actionable PGx ^a^ Consider reduction in starting dose for patients homozygous for the UGT1A1*28 allele. The precise dose reduction is not known and subsequent dose modifications should be considered based on individual patient tolerance to treatment [170]	-	-	-	Actionable PGx ^a^ Start with 70% of standard dose. If the patient tolerates it, the dose can be increased, guided by the neutrophil count [168]
Cetuximab/panitumumab	*EGFR* *KRAS* *NRAS*	Testing required ^b^ Determine *EGFR* expression status and confirm the absence of an *RAS* mutation before treatment [170]	-	-	Testing required ^b^ Test *RAS* status (*KRAS* and *NRAS* exons 2, 3 and 4) before treatment [171]	-

a: Actionable PGx—it may inform about changes in efficacy, dosage, metabolism or toxicity due to gene/protein/chromosomal variants or phenotypes, or contraindicate a drug in a subset of patients with particular variants/genotypes/phenotypes, without requiring prior testing. b: Testing required—it states that testing should be conducted before using a drug. This requirement may only be for a subset of patients.
